# Desensitization to Fear-Inducing COVID-19 Health News on Twitter: Observational Study

**DOI:** 10.2196/26876

**Published:** 2021-07-16

**Authors:** Hannah R Stevens, Yoo Jung Oh, Laramie D Taylor

**Affiliations:** 1 Department of Communication University of California, Davis Davis, CA United States

**Keywords:** desensitization, death toll, pandemic, fear-inducing, fear, health news, anxiety, COVID-19, mass media, public health, behavior change, coronavirus

## Abstract

**Background:**

As of May 9, 2021, the United States had 32.7 million confirmed cases of COVID-19 (20.7% of confirmed cases worldwide) and 580,000 deaths (17.7% of deaths worldwide). Early on in the pandemic, widespread social, financial, and mental insecurities led to extreme and irrational coping behaviors, such as panic buying. However, despite the consistent spread of COVID-19 transmission, the public began to violate public safety measures as the pandemic got worse.

**Objective:**

In this work, we examine the effect of fear-inducing news articles on people’s expression of anxiety on Twitter. Additionally, we investigate desensitization to fear-inducing health news over time, despite the steadily rising COVID-19 death toll.

**Methods:**

This study examined the anxiety levels in news articles (n=1465) and corresponding user tweets containing “COVID,” “COVID-19,” “pandemic,” and “coronavirus” over 11 months, then correlated that information with the death toll of COVID-19 in the United States.

**Results:**

Overall, tweets that shared links to anxious articles were more likely to be anxious (odds ratio [OR] 2.65, 95% CI 1.58-4.43, *P*<.001). These odds decreased (OR 0.41, 95% CI 0.2-0.83, *P*=.01) when the death toll reached the third quartile and fourth quartile (OR 0.42, 95% CI 0.21-0.85, *P*=.01). However, user tweet anxiety rose rapidly with articles when the death toll was low and then decreased in the third quartile of deaths (OR 0.61, 95% CI 0.37-1.01, *P*=.06). As predicted, in addition to the increasing death toll being matched by a lower level of article anxiety, the extent to which article anxiety elicited user tweet anxiety decreased when the death count reached the second quartile.

**Conclusions:**

The level of anxiety in users’ tweets increased sharply in response to article anxiety early on in the COVID-19 pandemic, but as the casualty count climbed, news articles seemingly lost their ability to elicit anxiety among readers. Desensitization offers an explanation for why the increased threat is not eliciting widespread behavioral compliance with guidance from public health officials. This work investigated how individuals' emotional reactions to news of the COVID-19 pandemic manifest as the death toll increases. Findings suggest individuals became desensitized to the increased COVID-19 threat and their emotional responses were blunted over time.

## Introduction

### Background

The COVID-19 outbreak has spread worldwide, affecting most countries. Since the outbreak of COVID-19, the number of confirmed cases and the death toll have steadily risen. According to Johns Hopkins University, as of May 9, 2021, more than 157.9 million cases of COVID-19 and 3.2 million deaths have been reported worldwide [1]. Among the countries affected by COVID-19, the United States has had 32.7 million cases (23.5% of confirmed cases worldwide) and 580,000 deaths (17.7% of deaths worldwide). The overabundance of information, misinformation, and disinformation surrounding COVID-19 on social media in the United States has fueled a COVID-19 infodemic, which has jeopardized public health policy aimed at mitigating the pandemic [2], raising questions about the cognitive processes underlying public responses to COVID-19 health information.

Extreme safety precautions (eg, statewide lockdowns, travel bans) have impacted individuals’ physical and mental health in the United States. People experienced intense psychological frustration and anxiety regarding the virus and strict safety measures (eg, stay-at-home measures), especially during the early stages of the COVID-19 pandemic [3-5]. Social, financial, and mental insecurities have even led to extreme and irrational coping behaviors, such as panic buying from January to March 2020 [5]. However, throughout the pandemic, the public became desensitized to reports of COVID-19’s health threat, and the rising number of confirmed cases and death toll began to lose impact [6,7]. As a result, segments of the public began violating public safety measures as the pandemic progressed, despite the consistent spread of COVID-19 [8-10].

From such observations, two key considerations arise. First, fear-eliciting health messages have a significant effect on eliciting motivation to take action to control the threat. However, repeated exposure to these messages over long periods results in desensitization to those stimuli. In this work, we examine the effect of fear-inducing news articles on people’s expression of anxiety on Twitter. Additionally, we investigate how people are desensitized by fear-inducing news articles over time, despite the steadily rising COVID-19 death toll.

### Effect of Fear-Inducing Messages on Public Anxiety

The current pandemic has fueled rapidly evolving news cycles and shaped public sentiment [11,12]. Public health experts’ recommendations to mitigate the COVID-19 threat, including widespread business shutdowns and physical distancing guidelines, have proven psychologically and emotionally taxing [13], inducing intense psychological frustration and anxiety among the public [3-5]. Previous literature suggests that fear-inducing messages influence emotions and behaviors when individuals perceive the message to be relevant (ie, they feel susceptible to the threat) and serious (ie, the threat is severe). That is, the heightened threat induces fear and anxiety that, in turn, motivate people to take action [14-17].

In the context of the COVID-19 pandemic, the efficacy of fear-inducing messages on behavioral compliance with public health officials is consequential. Reports of increased COVID-19 transmission and the rising death toll may elicit anxiety about the virus, consequently motivating behaviors intended to manage the problem. For instance, a national survey examining the mental health consequences of COVID-19 fear among US adults during March 2020 found that respondents generally expressed moderate to high COVID-19 fear and anxiety (7 on a scale of 10), and increased anxiety was most prevalent in areas with the highest reported COVID-19 cases [3]. Subsequently, the fear and anxiety induced by COVID-19–related threats can lead people to seek more health-related protective strategies. For example, one study found that as the threat of COVID-19 increased, people expressed more fear-related emotions and they were subsequently increasingly motivated to search for preventative behaviors and information online [5].

### Desensitization to Fear-Inducing Messages

Although fear-based health messages have been shown to motivate changes in behavior, repeated exposure to even highly arousing stimuli—such as news of the rising death toll from COVID-19—may eventually result in desensitization to those stimuli [6,18]. Desensitization refers to the process by which cognitive, emotional, and physiological responses to a stimulus are reduced or eliminated over protracted or repeated exposure [19]. It can play an important adaptive role in allowing individuals to function in difficult circumstances that might otherwise result in overwhelming and persistent anxiety or fear. For example, one analysis of Twitter messages from a region of Mexico with then-rising violence found the expressions of negative emotions declined [20]. Although increasing anxiety and fear might prompt security-seeking behavior, these emotions may also be paralyzing; some measure of desensitization can facilitate continuing with necessary everyday tasks.

Numerous studies have demonstrated desensitization to media content. Research has often focused on fictional depictions of violence [21,22]; however, desensitization has also been demonstrated in response to repeated exposure to violent news stories [23], hate speech [24], and sexually explicit internet content [25], although this last finding has mixed support [26].

Researchers studying social media data have explored the possibility that news messages can result in desensitization. Li and colleagues [27] analyzed a large sample of Twitter data, examining posts linked to guns and shootings for emotional language. They observed that across 3 years of mass shootings and school shootings in the United States, the frequency of negative emotional words used in shooting-related tweets declined; they argued that this reflected desensitization to gun violence.

In the context of the COVID-19 pandemic, news audiences have been repeatedly exposed to highly arousing messages related to COVID-19–related deaths—messages that inherently communicate explicit and implicit threats of serious illness and death to readers. Fundamentally, the biological response to threat communicated through text is similar to threats communicated in other ways [28]. Over time, as the death toll has increased, the cognitive, emotional, and physiological responses to threatening COVID-19 news may have been blunted. Individuals may have become desensitized to threatening COVID-19 information and experienced diminished anxiety over time, even in the face of an increasing threat.

### Rationale and Aims

The public relies heavily on news disseminated through social media for information about the spread of the virus [29]. Twitter, in particular, is a popular outlet for sharing news [30] and has become a forum for individuals to communicate their feelings about COVID-19 [11]. Social media text analysis has emerged as a particularly effective way to assess sentiment dynamics surrounding public health crises; consider, for example, the Zika outbreak [31]. This study uses social media text analysis to examine the anxiety levels in news articles and related tweets over 11 months, then considers those levels in the context of deaths from COVID-19 on the day the post was shared [32].

The general hypothesis guiding this research is that audiences will have become desensitized to COVID-19 deaths over the course of the pandemic, decreasing the level of anxiety elicited by fearful COVID-19 health information reported in the news. To the best of our knowledge, ours is the first study to investigate whether, as the objective threat and harm of COVID-19 has increased, individuals have become desensitized to news reports of cautionary COVID-19 health information.

## Methods

### Overview

This study examined how anxiety levels in news articles predicted users’ tweet anxiety levels over 11 months, then correlated that information with the total death toll of COVID-19 in the United States as reported to the Centers for Disease Control and Prevention (CDC) on the day the post was shared [32]. Employing semantic analysis procedures to analyze anxiety in the full news articles and their corresponding user tweets allowed us to examine how fear elicited by COVID-19 health news manifests as individuals become desensitized to news of COVID-19–related deaths.

### Data Collection

**Figure 1:**
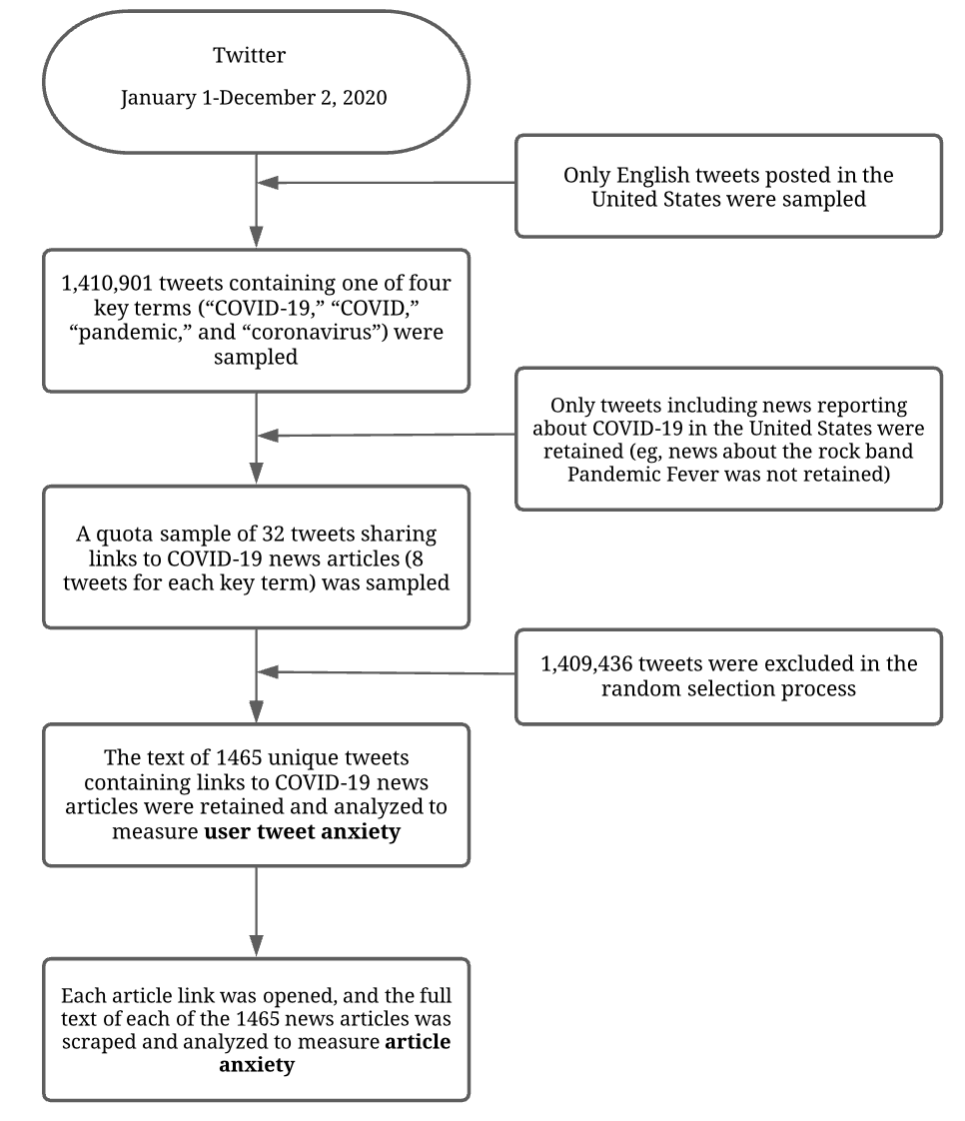
Flowchart of data collection process.

The sample comprises content shared to Twitter, a popular social media platform used for sharing news [30]. The text of 1465 news articles and corresponding posts by users were collected from tweets containing the terms “COVID-19,” “COVID,” “pandemic,” and “coronavirus” from January 1 to December 2, 2020. For an overview of the data collection process, see Figure 1.

The Python programming language was used to extract posts sharing news reports of COVID-19 health information. We collected a quota sample of 32,000 US tweets containing one of four key terms (ie, COVID, COVID-19, coronavirus, pandemic) each week from January 1 to December 2, 2020. The GetOldTweets3 Python3 library was used to scrape tweets for the months of January-July 2020 [33]. Twitter’s application programming interface (version 2) was used to collect tweets from August-December 2020 [34].

Human coders then filtered through the sample of 1,410,901 tweets to randomly extract a quota of 8 original tweets per key term from each week sharing a news report about COVID-19. Data collection resulted in thousands of tweets containing links per week. To facilitate the representativeness of the news articles, 32 tweets were drawn from each week from a shuffled list of tweets containing hyperlinks. Since we aimed to assess users’ reactions to the text of the article they read, without the confounding textual framing of other peoples’ commentary about an article, retweets were excluded from the analysis. If a quota of 32 tweets each week (8 per key term) was not met, additional tweets were sampled for that week. Notably, the disease and pandemic were not commonly referred to as COVID-19 in early January; accordingly, three weeks did not have 8 tweets with the terms “COVID” and “COVID-19” per week.

The news articles were collected from links shared by Twitter users in general, regardless of who posted the tweet. We only included users sharing links to news articles regarding COVID-19 in the United States; all other content was excluded (eg, news about the rock band Pandemic Fever). If all posts for that week were excluded, another sample from that week was drawn. If a tweet linked to a news article that had been taken down, a replacement post was sampled from the same week. We then extracted the text from the news articles and their corresponding tweets. The final sample was comprised of n=1465 news-sharing tweets.

### Linguistic Inquiry and Word Count Sentiment Analysis

**Figure 2:**
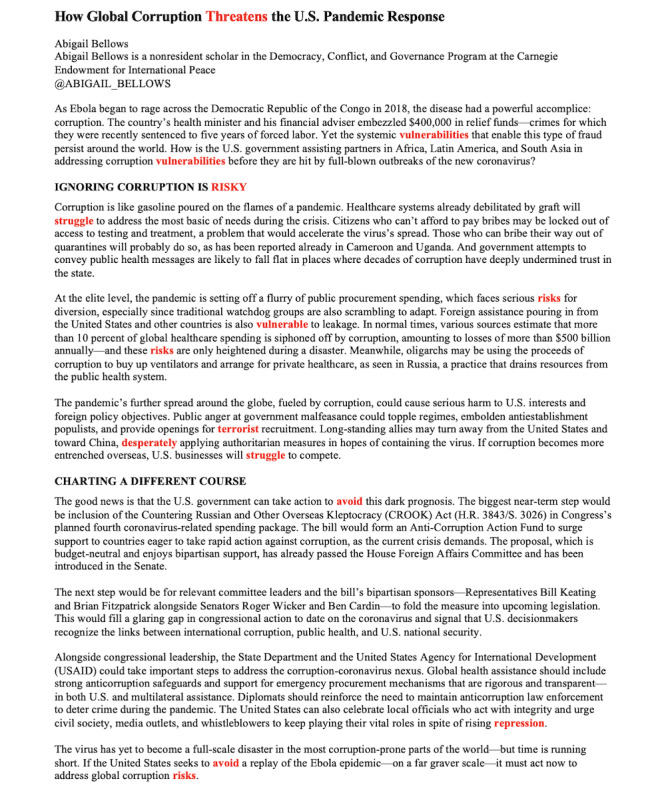
Sample text from a COVID-19 news article shared to Twitter [37]. The words highlighted in red are LIWC anxiety words. Since this article contains 15 anxiety words out of 745 words total (2.4%), this article is assigned a LIWC anxiety score of 2.4. LIWC: Linguistic Inquiry and Word Count.

Once the final sample was collected (n=1465), we analyzed articles and tweets using the Linguistic Inquiry and Word Count (LIWC) program [35]. The body text of the news articles was analyzed to measure article anxiety, while the tweet text was analyzed to measure tweet anxiety. LIWC is a natural language processing text analysis program that classifies texts by counting the percentage of words in a given text that fall into prespecified categories, such as a linguistic category (eg, prepositions) or psychological processes (eg, anxiety, sadness). In this study, we focused on the percentage of LIWC anxiety lexicon words in news articles and tweets because this psychological process is germane to the efficacy of fear-based news messaging [14,36]. LIWC calculates the percentage of anxiety words relative to all words contained in a text to account for long versus short text classification. For example, we might discover that 15/745 (2.04%) words in a given article were anxiety lexicon words. The LIWC output would then assign that particular article an anxiety score of 2.04 (see Figure 2 for an example).

### Statistical Analysis

We paired the final sample with the CDC’s aggregate death toll on the day the tweet was posted. Contextualizing the articles and tweets allowed us to examine how fear elicited by COVID-19 health news manifests as individuals become desensitized to news of COVID-19–related deaths.

The outcome of interest was tweet anxiety. Note that the distribution of count data outcome variables (in our case, LIWC tweet anxiety) often contains excess zeros; this result is known as zero inflation. The positive values are skewed, and a considerable “clumping at zero” is trailed by a bump representing positive values [38]. In our specific distribution, the “clumping at zero” represents texts containing zero anxiety lexicon terms. Generalized linear models are not appropriate for zero-inflation data. As all observed zeros are unambiguous, they are best analyzed separately from the nonzeros.

Two distinct distributions generally characterize zero-inflation data; thus, a zero-inflated model, which separates the zero and nonzero counts, is appropriate [39,40]. In zero-inflated models, the distribution of positive count values depends on the probability of exceeding the hurdle and reaching the distribution of positive values. In other words, it considers the odds of having any anxiety in a tweet versus none at all. For tweets that clear the hurdle, it then considers how much anxiety will be in a tweet on a continuous distribution.

**Figure 3:**
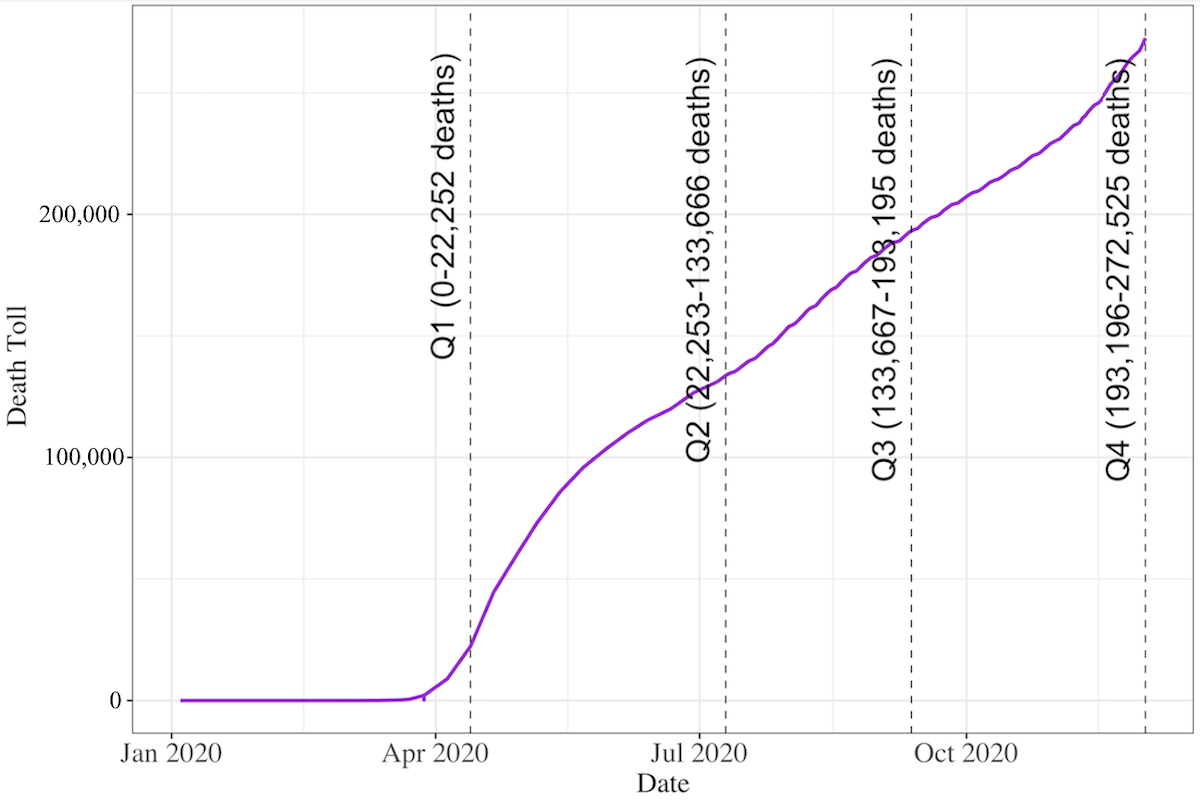
Distribution of death toll quartiles over time.

We employed a zero-inflated model using a gamma distribution with a log link to examine any association between article anxiety and death toll, along with their interaction with subsequent tweet anxiety for all values of tweet anxiety greater than zero. We paired that with a model that used a binomial distribution with a logit link to determine zero anxiety versus nonzero anxiety in tweets. We recoded the death toll into categories reflecting the death count at the second quartile, the third quartile, and the fourth quartile relative to the first quartile of the total death count (see Figure 3 for a breakdown). This was necessitated by the skewed and logarithmic character of the distribution. These values were then used in place of the continuous variable to model the interaction. We used R statistical software for data analysis (version 3.6.2; The R Foundation for Statistical Computing).

### Ethics Statement

This study only used information available in the public domain. No personally identifiable information was included in this study. Ethical review and approval was not required for this study because the institutional review board recognizes that the analysis of publicly available data does not constitute human subjects research.

## Results

**Figure 4:**
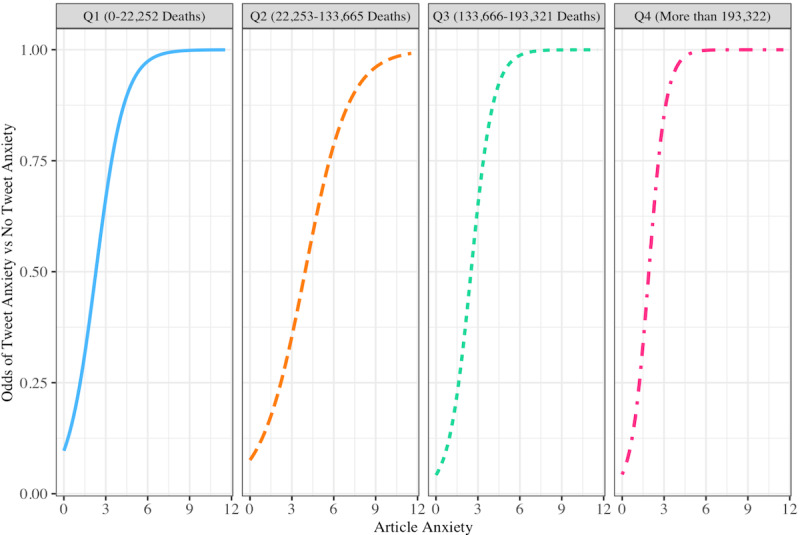
Article anxiety predicting the odds of tweet anxiety versus no tweet anxiety at the first, second, third, and fourth quartiles of the COVID-19 death toll.

Results suggest that as the death toll increased over time, the baseline level of anxiety lexicon words in articles decreased; this was evidenced by our finding that when the pandemic’s severity and threat increased, individuals shared less news coverage containing COVID-19 anxiety words (eg, “risk,” “worried,” “threatens”). When assessing the odds of a tweet having no anxiety versus anxiety, we found that the baseline odds of *not* having anxiety in a tweet were 0.11; the odds of having anxiety in a tweet increased (odds ratio [OR] 2.65, 95% CI 1.58-4.43, *P*<.001) with each unit increase in anxiety within an article. The odds of tweet anxiety decreased as paired with CDC total deaths in the third quartile (OR 0.41, 95% CI 0.2-0.83, *P*=.01) and fourth quartile (OR 0.42, 95% CI 0.21-0.85, *P*=.01), respectively (see Table 1 and Figure 4).

**Table 1:** The odds of a tweet containing anxiety language versus no anxiety language, as determined using a zero-inflated model with categorical death^a^.

Variable	Odds ratio (95% CI)	*P* value
Intercept	0.11 (0.07-0.16)	<.001
Anxiety in article	2.65 (1.58-4.43)	<.001
Second quartile (22,253-133,665 deaths)	0.76 (0.41-1.41)	.39
Third quartile (133,666-193,321 deaths)	0.41 (0.2-0.83)	.01
Fourth quartile (≥193,322 deaths)	0.42 (0.21-0.85)	.02
Interaction anxiety in article by second quartile deaths (22,253-133,665 deaths)	0.71 (0.34-1.48)	.36
Interaction anxiety in article by third quartile deaths (133,666-193,321 deaths)	1.32 (0.54-3.24)	.55
Interaction anxiety in article by fourth quartile deaths (≥193,322 deaths)	1.9 (0.75-4.83)	.18

^a^This table reports the odds of no tweet anxiety versus tweet anxiety. Deaths were categorized based on the second, third, and fourth quartiles relative to the first quartile.

**Figure 5:**
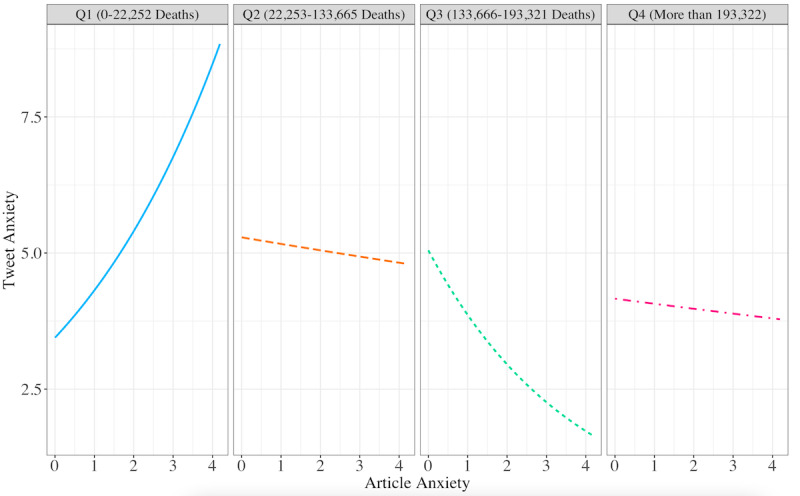
Article anxiety predicting nonzero tweet anxiety at the first, second, third, and fourth quartiles of the COVID-19 death toll.

We then examined the actual estimated linguistic anxiety of tweets, looking only at all of the values in a continuous distribution, excluding those values with zero anxiety (ie, the tweet did not contain any anxiety lexicon words). Although not statistically significant at *P*<.05, the results illuminate an emerging yet meaningful trend. The baseline level of anxiety in a tweet was 3.45. The tweet anxiety level trend increased (OR 1.25, 95% CI 0.99-1.59, *P*=.068) with each unit increase of article anxiety. Overall, tweets that shared links to more anxious articles expressed more anxious terms (eg, “avoid,” “uncertain,” “paranoid”). Notably, the interaction between article anxiety and deaths was not found to be a significant predictor of tweet anxiety level. Tweet anxiety rose rapidly with articles when the death toll was low and then decreased in the third quartile of deaths (OR 0.61, 95% CI 0.37-1.01, *P*=.06). As predicted, in addition to the increasing death toll being matched by a lower level of article anxiety, the extent to which article anxiety elicited tweet anxiety decreased when the death count reached the second quartile (see Table 2 and Figure 5).

**Table 2:** Actual anxiety expressed in tweets, as predicted by article anxiety and COVID-19 death toll: gamma regression model with categorical death^a^.

Variable	Coefficient (95% CI)	*P* value
Intercept	3.45 (2.77-4.28)	<.001
Anxiety in article	1.25 (0.99-1.59)	.07
Second quartile (22,253-133,665 deaths)	1.53 (1.1-2.15)	.01
Third quartile (133,666-193,321 deaths)	1.47 (0.97-2.22)	.17
Fourth quartile (≥193,322 deaths)	1.21 (0.83-1.75)	.32
Interaction anxiety in article by second quartile deaths (22,253-133,665 deaths)	0.78 (0.56-1.08)	.14
Interaction anxiety in article by third quartile deaths (133,666-193,321 deaths)	0.61 (0.37-1.01)	.06
Interaction anxiety in article by fourth quartile deaths (≥193,322 deaths)	0.78 (0.5-1.22)	.28

^a^Deaths were categorized based on the second, third, and fourth quartiles relative to the first quartile. This table reports the actual estimated anxiety in the tweet, looking only at all of the values in a continuous distribution, excluding those with zero anxiety.

## Discussion

### Principal Findings

This study reports exploratory findings on the effects of fear-inducing news messages during a pandemic. Most importantly, we demonstrated a link between the anxiety expressed in news articles and the odds of anxiety being expressed by those who shared the articles to Twitter. This likely reflects the ability of pandemic-related news messages to elicit a measure of fear in their readers, consonant with public health goals. However, likely as a function of the rising COVID-19 threat over time (as indicated by LIWC news article anxiety) and a low perceived ability to prevent the rapid spread of the virus, anxiety did not increase in response to climbing death tolls over time. Instead, anxiety in tweets increased sharply in response to article anxiety early on in the pandemic, but as the death toll climbed, it flattened out, and news articles seemingly lost their ability to elicit anxiety among readers.

Such findings from this study provide several insights and directions for future research. Our findings reveal that responses to COVID-19 news as well as the rising death toll are increasingly bland. Growing desensitization in the face of threatening pandemic information impedes public health experts’ efforts to mitigate the COVID-19 crisis [41]. Therefore, future research should investigate how to “resensitize” the public and motivate them to take active roles in COVID-19–related responses (eg, wearing masks, washing hands, vaccination). Here, literature on behavioral theories may be helpful in implementing effective resensitization tactics. For instance, the transtheoretical model [42,43], which explains behavior change through stages of change, suggests that to initiate and maintain health behaviors, it is important to have supportive relationships and motivate one another to share successes and experiences related to engaging in certain behaviors. In addition, it is suggested that reinforcement management—such as getting rewards from behavioral engagement—can be effective. In the context of COVID-19, health care providers can apply these tactics (ie, social support, reward) to motivate people to adhere to public health measures such as vaccination.

Second, since extant research shows that both statistics (eg, percentage of deaths) and cognitive dissonance can elicit desensitization [44,45], scholars should investigate the role of additional psychological processes in desensitization to the COVID-19 threat. Third, as self-disclosure varies by platform [46], more work is needed to explore how anxiety manifests on other platforms for discussing COVID-19 news. Finally, our findings suggest that health care practitioners should be prepared for public desensitization to future global pandemic scenarios. More specifically, it would be important to carefully monitor the public’s level of desensitization to health news and implement appropriate resensitization strategies based on different stages in the pandemic.

### Limitations

Our findings illuminate desensitization to fear-inducing news messages during the pandemic; however, this study is not without limitations. By focusing on Twitter, we neglected to explore how anxiety manifests on other platforms for sharing news (eg, the comments section of digital news sites). As different platforms have different community norms [46], it is reasonable to expect manifestations of anxiety to vary by platform. Furthermore, Twitter users are younger, more democratic, and wealthier than the general population of Americans [47]. Acknowledging the biases associated with using computational social media data [48], our findings should be interpreted as representing a subset of the US population (ie, Twitter users), not all US residents. Second, among 1.4 million tweets collected, only a small number of tweets were sampled in this study. Therefore, our study may lack generalizability. Additionally, the LIWC computerized coding tool does not allow for the nuanced coding that could be achieved with human coders. Although we have attempted to minimize this potential bias using a well-validated sentiment analysis procedure, LIWC [35], this study is limited in its use of anxiety in text as a measure of user anxiety.

### Conclusions

This work investigates how individuals' emotional reactions to news of the COVID-19 pandemic manifest as the death toll increases. Individuals become desensitized to an increased health threat and their emotional responses are blunted over time. Our results suggest desensitized public health reactions to threatening COVID-19 news, which could affect the propensity of individuals to adopt recommended health behaviors.

Public health agencies made recommendations to slow the pandemic’s spread, including physically distancing from others when appropriate, wearing masks, engaging in frequent handwashing, and disinfecting frequently touched surfaces. The consequences of ignoring these guidelines initially incited widespread fear and anxiety around contracting the virus or having family and friends contract it and fall ill. Social scientists have tried to inform interventions aimed at promoting compliance with public health experts [49]. The results of this study suggest the increased threat conveyed in COVID-19 news has, however, diminished public anxiety, despite an increase in COVID-19–related deaths. Desensitization offers one way to explain why the increased threat is not eliciting widespread compliance with guidance from public health officials. This work sheds light on both the effectiveness and shortcomings of fear-based health messages during the pandemic, as well as the utility of natural language processing to gain an understanding of public responses to emerging health crises.

## Abbreviations

CDC: Centers for Disease Control and Prevention
LIWC: Linguistic Inquiry and Word Count
OR: odds ratio
