# Metadata Correction: “Desensitization to Fear-Inducing COVID-19 Health News on Twitter: Observational Study”

**DOI:** 10.2196/32231

**Published:** 2021-07-28

**Authors:** Hannah R Stevens, Yoo Jung Oh, Laramie D Taylor

**Affiliations:** 1 Department of Communication University of California, Davis Davis, CA United States

In “Desensitization to Fear-Inducing COVID-19 Health News on Twitter: Observational Study” (JMIR Infodemiology 2021;1(1):e26876) the authors noted one error.

One author's name was displayed as follows:

Laramie R Taylor

The middle initial in the name has now been corrected as follows:

Laramie D Taylor

The correction will appear in the online version of the paper on the JMIR Publications website on July 28, 2021, together with the publication of this correction notice. Because this was made after submission to PubMed, PubMed Central, and other full-text repositories, the corrected article has also been resubmitted to those repositories.

